# Stage-Dependent Changes of Visual Function and Electrical Response of the Retina in the *rd10* Mouse Model

**DOI:** 10.3389/fncel.2022.926096

**Published:** 2022-07-19

**Authors:** Seongkwang Cha, Jungryul Ahn, Yurim Jeong, Yong Hee Lee, Hyong Kyu Kim, Daekee Lee, Yongseok Yoo, Yong Sook Goo

**Affiliations:** ^1^Department of Physiology, Chungbuk National University School of Medicine, Cheongju, South Korea; ^2^Department of Biochemistry, Chungbuk National University School of Medicine, Cheongju, South Korea; ^3^Department of Microbiology, Chungbuk National University School of Medicine, Cheongju, South Korea; ^4^Department of Life Science, Ewha Womans University, Seoul, South Korea; ^5^Department of Electronics Engineering, Incheon National University, Incheon, South Korea

**Keywords:** retinal ganglion cell, retinal degeneration, retinal prosthesis, optomotor response, multichannel recording

## Abstract

One of the critical prerequisites for the successful development of retinal prostheses is understanding the physiological features of retinal ganglion cells (RGCs) in the different stages of retinal degeneration (RD). This study used our custom-made *rd10* mice, C57BL/6-*Pde6b^em1(R560C)Dkl^*/Korl mutated on the Pde6b gene in C57BL/6J mouse with the CRISPR/Cas9-based gene-editing method. We selected the postnatal day (P) 45, P70, P140, and P238 as representative ages for RD stages. The optomotor response measured the visual acuity across degeneration stages. At P45, the *rd10* mice exhibited lower visual acuity than wild-type (WT) mice. At P140 and older, no optomotor response was observed. We classified RGC responses to the flashed light into ON, OFF, and ON/OFF RGCs *via in vitro* multichannel recording. With degeneration, the number of RGCs responding to the light stimulation decreased in all three types of RGCs. The OFF response disappeared faster than the ON response with older postnatal ages. We elicited RGC spikes with electrical stimulation and analyzed the network-mediated RGC response in the *rd10* mice. Across all postnatal ages, the spikes of *rd10* RGCs were less elicited by pulse amplitude modulation than in WT RGCs. The ratio of RGCs showing multiple peaks of spike burst increased in older ages. The electrically evoked RGC spikes by the pulse amplitude modulation differ across postnatal ages. Therefore, degeneration stage-dependent stimulation strategies should be considered for developing retinal prosthesis and successful vision restoration.

## Introduction

Retinal prostheses for the blinds have received considerable attention. Patients with retinitis pigmentosa (RP) lose sight due to a gradual loss of photoreceptors in the outer nuclear layer (ONL) (Hartong et al., 2006). On the other hand, inner retinal neurons such as bipolar cells (BCs) or retinal ganglion cells (RGCs) remain preserved compared with photoreceptors (Stone et al., 1992; Kim et al., 2002; Mazzoni et al., 2008). For patients with RP, retinal prostheses have successfully elicited phosphenes by electrically activating inner retinal neurons in the degenerate retina (Stingl et al., 2015; Luo and da Cruz, 2016). However, retinal prostheses have limited performance in clinical trials, resulting in poor visual acuity (less than 20/420) in device-implanted patients (Ayton et al., 2020; Im and Kim, 2020).

Among genetic models for human RP, the retinal degeneration (RD) 10 (*rd10*) mouse is a widely used animal model due to their delayed onset and slower progression of RD than *rd1* mice (Chang et al., 2002; Chang et al., 2007). Although the afferent nerve connection between the retina and the brain is maintained, histological and physiological changes appear in the remaining neurons with the progress of RD. The progress of RD distinguishes into three phases: Phase I: rod degeneration, Phase II: cone degeneration, and Phase III: remodeling of the remnant retinal network (Marc et al., 2003). Age-dependent histological changes were well investigated in the *rd10* mouse retinal neurons such as rods, cones, BCs, horizontal cells (HCs), amacrine cells (ACs), and RGCs (Chang et al., 2007; Gargini et al., 2007; Barhoum et al., 2008; Mazzoni et al., 2008; Phillips et al., 2010; Murakami et al., 2012; Pennesi et al., 2012; Li et al., 2019). In *rd10* mice, RD initiates around Postnatal day (P)18–20 due to the progressive death of rod photoreceptors, and the rod photoreceptor loss lasts until P45–70. Although the somas of cone photoreceptors preserve until P270–285, the degeneration of cone photoreceptors’ outer and inner segments occurs before P70. Twenty percent of rod bipolar cells (RBCs) decrease between P45 and P105. Thirty-nine percent of HCs decrease between P45 and P270. Nevertheless, RGCs do not show significant changes until nine months of age.

In contrast to these histological studies, electrophysiological studies regarding electrically evoked RGC responses according to the degeneration stages are still insufficient. A few studies reported the light-driven RGC response or electrically evoked RGC response up to P60 in *rd10* mice (Stasheff et al., 2011; Yoon et al., 2020). These studies use limited postnatal days since they first classified the RGC types based on the light-driven response, then followed up on the RGC type-specific electrical response. However, our previous study using up to P238 *rd10* mice focused on the changes of a burst pattern of RGCs. From P14 to P30, electrically evoked RGC spikes show a single burst like WT, while multiple bursts appear around P45, and the frequency of multiple bursts slows down after P70 (P70 to P238) (Goo et al., 2011b,^2016^; Park et al., 2015). Therefore, guided by histological and electrophysiological changes, we selected P30, P45, P70, P140, and P238 as representative ages for RD stages.

In this study, we observed the degeneration stage-dependent change of visual function and RGC responsiveness to light stimulation with our custom-made *rd10* mice, C57BL/6-*Pde6b^em1(R560C)Dkl^*/Korl mutated on the Pde6b gene in C57BL/6J mouse with CRISPR/Cas9-based gene-editing method. In addition, we investigated the degeneration stage-dependent change of network-mediated responses of RGCs to electrical stimulation.

## Materials and Methods

### Animals

This study was approved by the Institutional Animal Care and Use Committee of the Chungbuk National University (approval number: CBNUA-1520-21-01). All procedures followed the guidelines of the Association for Research in Vision and Ophthalmology Statement for the Use of Animals in Ophthalmic and Vision Research. In this study, we used newly established *rd10* mice (C57BL/6-*Pde6b^em1(R560C)Dkl^*/Korl strain). The *rd10* mice in a genetically heterozygous state were obtained from the National Institute of Food and Drug Safety Evaluation (NIFDS, Cheongju, South Korea). The heterozygotes were bred to produce the homozygotes. Among the pups, we selected only homozygotes and used them for this study. Remained homozygotes were bred with C57BL/6J mice (Raonbio, Yongin, South Korea) to maintain heterozygotes. All mice were housed under a 12-h day/night cycle under standard conditions. The wild-type (WT) mice (C57BL/6J mice) were used as control. We used adult WT mice (around P70), considering the maturation of retinal tissue. Furthermore, we used *rd10* mice of P30, P45, P70, P140, and P238, considering their degeneration stages based on histological studies (Gargini et al., 2007; Pennesi et al., 2012) and our previous study (Park et al., 2015; Goo et al., 2016). For enucleation of the eye, mice were anesthetized with an intramuscular injection of 30 mg/kg of tiletamine-zolazepam hydroxide (Zoletil 50; Virbac, Sao Paulo, Brazil), 10 mg/kg of xylazine hydrochloride (Rumpun; Bayer Korea, Seoul, South Korea), and 5,000 IU of heparin sodium (heparin; JW Pharmaceutical Corp., Seoul, South Korea).

### Generation and Genotyping of *rd10* Mouse

Using standard procedures, one-cell embryos were obtained from superovulated C57BL/6J (B6) female mice mated with B6 male mice. Cas9 mRNA and Pde6b sgRNA were prepared as described previously (Oh et al., 2021). The target sequence for Pde6b sgRNA is 5′- GCCGTGGCGCCAGTTGTGGTAGG-3′ (underline indicates PAM sequence) (Supplementary Figure 1A). The oligodeoxynucleotides (ODNs, 5′-CTAGCCCATC CAATTTACATACGTACCATGAGTAGGGTAAACATGGTCTG GGCTACATTGAAGCCGTGGCACCAATTATGATACGTGAT TCTTCGATAGGCTTTGCTGACAGAGAA-TAGAAAGCGCA CCAAGACCTGGGGAGCAGAGTAC-3′) contained codon 560 Arg (CGC) replaced by the Cys (TGC) sequence to introduce the desired knockin of mutation R560C with extra four silence mutations and overlapping the *Pde6b* exon 13 region for homologous recombination. We microinjected a mixture of 20 μg/ml sgRNA, 100 μg/ml ODNs, and 10 μg/ml Cas9 mRNA in 10 mM Tris-HCl, pH 7.4, and 0.1 mM EDTA into the pronucleus of one-cell eggs and transferred survived one-cell embryos into the pseudopregnant ICR females. Initial screening of F0 knockin mutants was analyzed by agarose gel electrophoresis of PCR product after restriction digestion with HhaI (Supplementary Figure 1B). Only mutant mice were further crossed with B6 mice, and the exact sequence of knockin mutation was verified by sequencing after TA cloning of PCR product from F1 heterozygous mice (Supplementary Figure 1C). The heterozygotes mice were maintained by backcrossing with B6 mice for 2 to 3 more generations before interbreeding.

**FIGURE 1:**
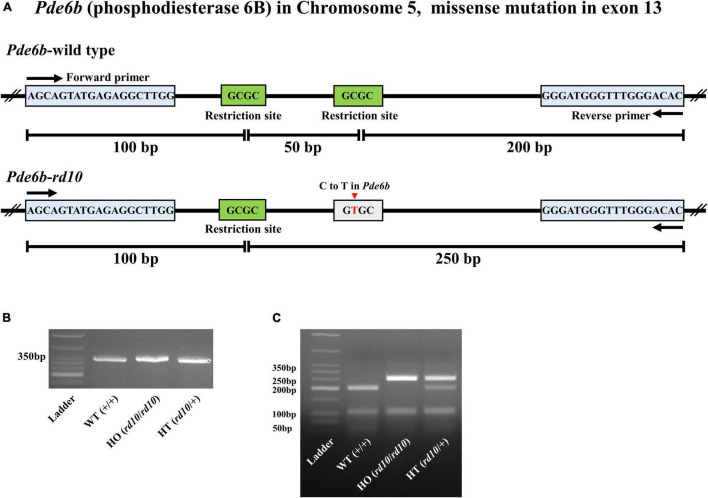
Genotyping of C57BL/6-*Pde6b *^em1(R560C)Dkl^**/Korl knockin mice. **(A)** The structures of the *Pde6b*-wild type allele and *Pde6b*-*rd10* allele are shown. Mutation point exists in exon 13 of rod phosphodiesterase gene (*Pde6b*) located on chromosome five of wild-type (WT) mouse (C57BL/6J). The mutation point (C to T in *Pde6b*) is presented by the red arrow. The primer binding sites are represented by the blue box. The restriction sites of Hha I are represented by the green box. **(B)** All PCR products of *Pde6b* from three different mice, wild-type (WT) (+/+), HO (*rd10/rd10*), and HT (*rd10*/+), have the same size as 350bp. **(C)** Digestion of PCR products with Hha I yielded 3, 2, and 4 DNA fragments in WT (+/+), HO (*rd10*/*rd10*), and HT (*rd10*/+) samples, respectively.

To routinely genotype the pups of *rd10* heterozygotes, we modified PCR primers as shown in Figure 1A. We extracted genomic DNA from the tail end of mice under P28. Briefly, 1 mm of the tail was incubated for 30 min at 95°C in an alkaline lysis buffer [25 mM NaOH (Sigma, Saint Louis, United States) and 0.2 mM EDTA (WELGENE, Gyongsan, South Korea), pH 12]. After lysis, the samples were cooled at room temperature for 3 min and then a neutralization buffer [40 mM Tris-HCl (BioShop Canada Inc, Burlington, ON, Canada), pH 5.0] was added in a 1:1 ratio. The samples were centrifuged at 13,000 rpm for 2 min to separate only supernatants containing genomic DNA. After extracting genomic DNA, we amplified only 350 bp of PCR products, including the mutant point of exon 13 of the Pde6b gene (Figure 1A). The PCR was performed with 1 μg of the supernatant containing genomic DNA, and Maxime™ PCR PreMix (i-StarTaq, iNtRON Biotechnology, Seongnam, South Korea), primer mixture 10 pmol (Forward: 5′-AGCAGTATGAGAGGCTTGGA-3′ and Reverse: 5′-GTGTCCCAAACCCATCCCTT-3′) according to the following protocol: 1) initial denaturation at 95°C for 5 min, 2) 35 cycles of denaturation at 94°C for 30 s, annealing at 61°C for 30 s, and extension at 72°C for 1 min and final extension at 72°C for 5 min. PCR products were confirmed by electrophoresis in a 3% agarose gel (iNtRON Biotechnology, Seongnam, South Korea) (Figure 1B). Finally, to judge the allele, we digested the PCR products with 14 units of HhaI (New England Biolabs, Ipswich, MA, United States) restriction enzymes that recognize the sequence of 5′-GCGC-3′. The DNA fragments were confirmed by electrophoresis in a 3% agarose gel (Figure 1C).

### Immunohistochemistry

Four WT mice (at P70) and 20 *rd10* mice (at P30, P45, P70, P140, and P238) were used for immunohistochemistry. We took 30-μm retinal sections in the 4% agarose embedding (Sigma–Aldrich, Saint Louis, MO, United States) after fixation with ice-cold 4% paraformaldehyde (PFA) (Biosesang, Seongnam, South Korea). The retinal sections were blocked with 4% normal goat serum (NGS) (Vector Laboratories, Inc., Burlingame, CA, United States) in 0.5% Triton X-100 (Sigma–Aldrich, Saint Louis, MO, United States) solution for 12 h, followed by incubation with each primary antibody at 4°C overnight. The primary antibodies were anti-rabbit opsin antibody, Red/Green (1:200, #AB5405, Merck Millipore, Saint Louis, MO, United States), anti-mouse rhodopsin antibody (1:200, #O4886, Merck Millipore, Saint Louis, MO, United States), and anti-mouse glutamine synthetase (GS) antibody (1:200, #MAB302, Merck Millipore, Saint Louis, MO, United States). After incubation with primary antibodies, the secondary antibodies were treated in the retinal sections at 4°C for 2 h. The secondary antibodies were Alexa Fluor^®^ 488 AffiniPure Goat Anti-Mouse IgG (1:500, #111-545-146, Jackson ImmunoResearch Inc., West Grove, PA, United States) and Alexa Fluor^®^ 647 AffiniPure Goat Anti-Rabbit IgG (1:500, #111-605-144, Jackson ImmunoResearch Inc., West Grove, PA, United States). The fluorescence image was captured using confocal microscopy (LSM800, Zeiss, Jena, Germany).

### Optomotor Response

**FIGURE 2:**
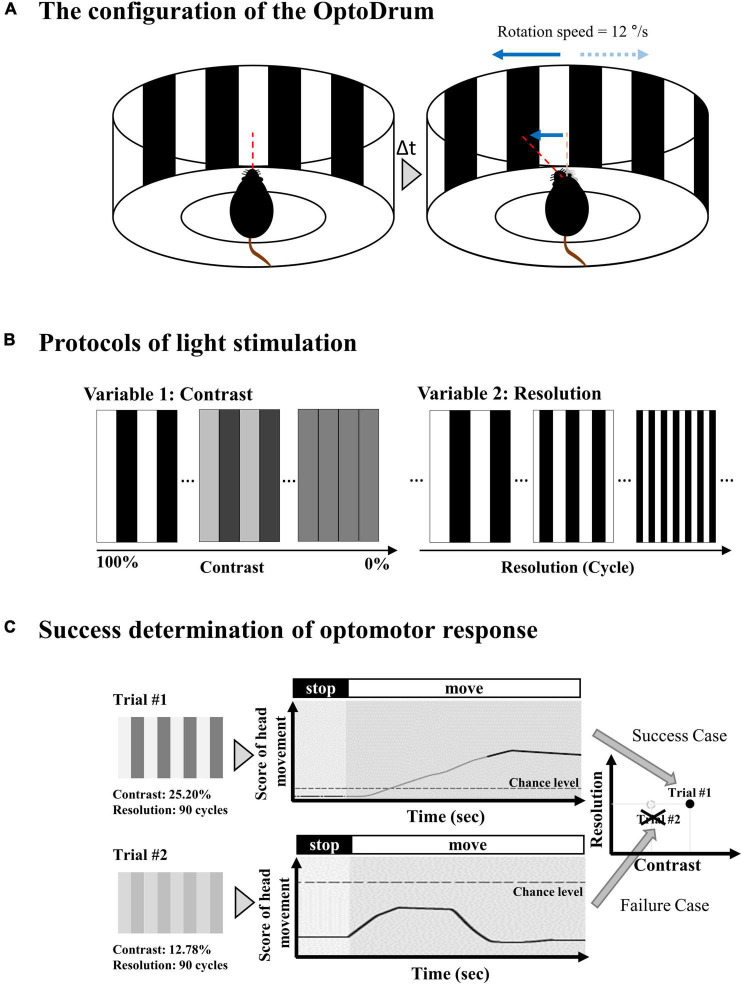
Experimental setup for optomotor response. **(A)** The mouse is located in a virtual rotating drum center with a black and white stripe. The drum rotates clockwise or counterclockwise with 12°/s of velocity. **(B)** The stripe pattern of the virtual rotating drum can be modulated in terms of relative contrast between black and white bar and spatial resolution. The contrast changes from 100% (black = #000000 (Hex code color of RGB) and white = #FFFFFF) to 0% (black = #808080 and white = #808080). The spatial resolution changes from 0 cyc/° (cycles per degree) to 0.7 cyc/°. **(C)** Among experimental trials, the successful trial of optomotor response is determined if the head movement score exceeds the chance level of the stimulus-independent head motion.

**FIGURE 3:**
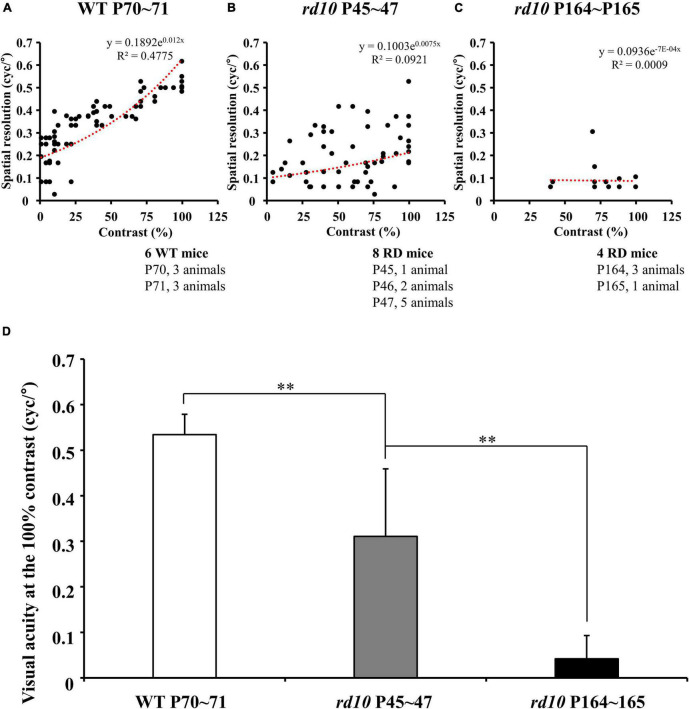
The progressive decrease of visual acuity in *rd10* mice. **(A–C)** The highest spatial resolution that induces the mouse’s optomotor response was measured under various contrast levels in wild-type (WT) (at P70–71) and *rd10* mice (at P45–47 and P164–165). The highest spatial resolution measured from subjects within the same group was plotted on the graph without distinguishing the individuals. The linear regression line was fitted using an exponential function (red dashed line). Among old *rd10* mice (P164–165), half animals did not show any optomotor response in this task. **(D)** The average visual acuity (mean ± SD) in a WT group and two *rd10* groups were compared. The statistical analysis was performed with ANOVA, and *post hoc* analysis was performed with Tukey’s HSD and Duncan’s test (**p < 0.01).

The optomotor response was evaluated using the Optodrum (Striatech GmbH, Tübingen, Germany). The mouse was positioned on an elevated stage at the height of 14.5 cm, which was placed in the center of the Optodrum (Figure 2). The walls of the Optodrum consisted of four LCD monitors, and the ceiling and floor included mirrors. The stimulation presented on monitors formed a virtual rotating drum with the black-and-white stripe pattern (radiance = 2 W⋅sr^–1^⋅m^–2^). An automatically controlled tracking system centered the virtual rotating drum over the mouse’s head. The virtual drum rotates clockwise or counterclockwise with 12°/s of velocity. The stimulation was applied for at least 1 s and ended either after at most 7.5 s, or when the animal became restless and started walking around. Among experimental trials, the successful trial of optomotor response was determined if the head movement score exceeded the chance level of the stimulus-independent head movement, which the algorithm of Optodrum provided (Benkner et al., 2013). Regarding the two parameters, contrast and spatial resolution, when we fix the contrast level, the spatial resolution (cycles per degree) was changed and vice versa. All the successful trials at either the lowest contrast level or highest spatial resolution were plotted on the graph of contrast versus spatial resolution (Figure 3). From this graph, we drew the linear regression curve, representing the visual acuity. More details can be found in the manufacturer’s publication (Benkner et al., 2013).

### Electrophysiological Recording With Multi-Electrode Array

*In vitro* recording of RGC activities was performed as follows: The lens and vitreous body were removed from the extracted eyeball. The retina was isolated from the posterior structure of the eyeball and cut into approximately 5 mm × 5 mm, including the center of the retina. The flattened retinal patch was attached to the multi-electrode array (MEA) with the ganglion cell layer (GCL) facing down. Retinal preparation after removing the vitreous body was prepared under near-infrared illumination in an artificial cerebrospinal fluid (ACSF) solution (124 mM NaCl, 10 mM glucose, 1.15 mM KH2PO4, 25 mM NaHCO3, 1.15 mM MgSO4, 2.5 mM CaCl2, and 5 mM KCl; all from Millipore, Burlington, ON, United States) bubbled with 95% O2 and 5% CO2 to maintain a pH of 7.3∼7.4 at 25°C.

The retinal activities were extracellularly recorded using a 60-channel MEA recording system. Briefly, the MEA60 system (Multichannel Systems GmbH, Reutlingen, Germany) consists of an amplifier (MEA 1060-up), heating system (PH01 and TC01), peristaltic perfusion system (PPS2), data acquisition hardware (Mc_Card), plana perforated MEA (pMEA) (60pMEA200/30iR), and software (Mc_Rack). The retinal activities were recorded with a bandpass from 1 to 3,000 Hz, a gain of 1,200, and a sampling rate of 25 kHz. The pMEA has 59 titanium nitride (TiN) active electrodes in an 8 × 8 grid layout with an electrode diameter of 30 μm and inter-electrode distance of 200 μm on a porous polyimide foil isolator and a large internal reference electrode as channel 15. The four inactive channels are located at each corner of MEA to record an analog signal. Each active electrode has an impedance of 50 kΩ at 1 kHz. We continuously perfused with the oxygenated fresh ACSF to retinal tissue on the pMEA *via* small pores in the porous polyimide foil isolator using a peristaltic perfusion system (1–3 mL/min) during recording. The retinal activities were recorded after waiting 20 min for the stabilization of retinal tissue attached to pMEA.

Flashed white full-field illumination (ON 4 s, OFF 4 s, 50 times) was applied to confirm the retinal activities to light. Light stimulation was applied after dark adaptation for about 20 min. Light stimulation was implemented through software based on Psychtoolbox using Matlab (MathWorks, Natick, MA, United States). The light stimulation pattern was projected by a projector (ep7122; Hewlett-Packard, Palo Alto, CA, United States) and focused on a photoreceptor layer of retinal tissue by passing through multiple lenses and compressing the size to 5 mm × 5 mm. We controlled the intensity of applied light stimulation with 2.0ND filters (NE220B; Thorlabs Inc., NJ, United States). The intensity of the full-field illumination was 40 μW/cm^2^ (light ON), and the intensity of the background illumination was 4.9 μW/cm^2^ due to the monitor’s backlight (light OFF). The light condition was mesopic. In rodents, the mesopic light condition could activate rod photoreceptors like the scotopic condition (Kelber, 2018).

Using a stimulus generator (STG 1004, Multichannel systems GmbH, Reutlingen, Germany), the current pulse train was delivered to the retinal preparation through 1 of 60 channels (mostly channel 44 in the middle of the MEA), with the remaining channels serving as recording electrodes. Stimulation consisted of symmetrical cathodic phase-1st biphasic pulses. Pulse duration was fixed at 500 μs/phase, and pulse amplitudes of 1, 5, 10, 20, 30, 40, and 50 μA/phase were applied. Biphasic current pulses were applied 50 times once per second (1 Hz) for each pulse amplitude.

### Data Analysis

Retinal ganglion cell spikes were processed as follows. We sorted the timestamp of RGC spikes from the raw signal trace recorded by MEA. First, the raw trace was processed with a 100 Hz cut-off high-pass filter to eliminate the low-frequency components such as hum noise or local field potential (LFP) oscillating rhythm (Goo et al., 2011a). Subsequently, the filtered signal was processed with a spike sorting software (Offline Sorter™; Plexon Inc., Dallas, TX, United States) to separate multiunit activities containing different spike waveforms into individual cell units using principal component analysis (Lewicki, 1998). Finally, we isolated the timestamp of RGC spikes and analyzed them with commercial analysis software (NeuroExplorer^®^; Nex Technologies, Colorado Springs, CO, United States) and a custom-made MATLAB (MathWorks, Natick, MA, United States) codes.

The number of retinas used was three for WT and three for *rd10* per selected postnatal day (P45, P70, P140, and P238). With light stimulation, the number of RGCs harvested from WT was 322, and 310, 216, 315, and 310 at P45, P70, P140, and P238, respectively. Out of these RGCs, we classified the light-driven RGC responses as follows. The post-stimulus time histograms (PSTHs) with the light stimulus of 50 trials (recording time: 400 s, ON: 4 s, OFF: 4 s, 400 s/8 s = 50 trials) were obtained. When the RGC spike number within 100 ms after light stimulus onset, offset, and onset/offset in PSTH exceeds two times of spontaneous firing rates (marked by a red line), we classified the cell as ON, OFF, and ON/OFF type RGC, respectively (Masland, 2001; Stasheff, 2008; Stasheff et al., 2011; Baden et al., 2016) (Supplementary Figure 2).

With electrical stimulation, the number of RGCs harvested from WT was 198, and 298, 294, 229, and 205 at P45, P70, P140, and P238, respectively. Out of these RGCs, we defined the electrically evoked RGC responses as follows. First, we counted spontaneous RGC spikes, the average number of spikes in 25 s before the stimulation onset. Second, if RGC fires more than spontaneous spikes with electrical stimulation, the RGC was counted in the data pool. From the timestamp of RGC spikes, to consider only network-mediated RGC response, we eliminated spikes that appeared within 10 ms after stimulus onset to exclude the stimulus artifact or the direct response of RGC (Sekirnjak et al., 2006; Boinagrov et al., 2014; Ahn et al., 2017).

Then, we quantified electrically evoked RGC response for the time window of 100 ms and 500 ms and divided the responses into two groups based on the spike burst after stimulus onset in the PSTH or peri-stimulus raster plot. One group of RGCs showed a single peak of spike burst, while the other group of RGCs showed multiple peaks of spike burst.

For the group of RGCs showing multiple peaks of spike burst, the inter-peak frequency was estimated as follows: (1) The PSTH was calculated for each RGC using 50 electrical stimulations with the highest input current (50 μA). (2) The power spectral density of the PSTH was calculated using the fast Fourier transform. (3) The frequency with the largest power was defined as the dominant frequency.

Statistical analysis was performed using commercial software (IBM SPSS Statistics 24; International Business Machines Corporation, IBM, New York, NY, United States). A paired Student’s *t*-test was performed for statistical analysis between the two groups. An ANOVA analysis was performed with the *post-hoc* tests of Tukey’s HDS and Duncan’s test to determine the statistical difference among three and more groups.

## Results

### Progressive Outer Retinal Degeneration by Postnatal Ages in *rd10* Mice (C57BL/6-*Pde6b^em1(R560C)Dkl^*/Korl Strain)

**FIGURE 4:**
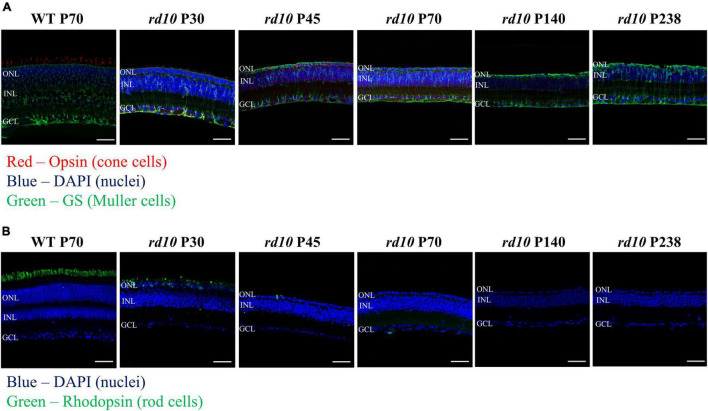
The progressive photoreceptor death by postnatal days in *rd10* mouse. **(A)** Opsin (red) and glutamate synthase (green) double labeling in the wild-type (WT) mice (C57BL/6J strain) at P70 and *rd10* mice (C57BL/6-*Pde6b^em1(R560C)Dkl^*/Korl strain) at P30, P45, P70, P140, and P238. **(B)** Immunostaining for rhodopsin (green) in WT mice and *rd10* mice. Nuclei are stained with DAPI (blue). Scale bars = 50 μm. ONL, outer nuclear layer; INL, inner nuclear layer; GCL, ganglion cell layer.

First, we validated the alteration of histological phenotypes by postnatal days in our custom-made *rd10* mice (C57BL/6-*Pde6b^em1(R560C)Dkl^*/Korl strain) to determine whether they are similar to the established *rd10* mice (B6.CXB1-*Pde6b^rd10^*/J strain) or not. Figure 4 shows visualized soma of retinal neurons, outer segments of photoreceptors, and cytosol of Müller cells. The ONL gradually decreases, leaving only a single layer of cell nucleus after P45 (Figure 4B). In addition, the fluorescence of outer segments of rod cells was not detected after P70, and the fluorescence of cone cells was not detected after P140 (Figure 4A). We also confirmed the gliosis by Müller cells. After P45, the Müller cells grew and sealed the photoreceptor and RGC layers.

### Progressive Decrease of Visual Acuity in *rd10* Mice by Postnatal Ages

The optomotor responses showed that the visual acuity of *rd10* mice was lower than that of WT mice although *rd10* mice were younger (at P45–47) than WT (at P70–71). For WT mice, the highest spatial resolution that induces the mouse’s optomotor response linearly increased as the contrast of the stripes increased (Figure 3A). On the other hand, for *rd10* mice (P45–47), the highest spatial resolution was highly variable and lower than WT mice (Figure 3B). Older mice (P164–165) rarely showed an optomotor response at all contrast levels (Figure 3C). At the 100% contrast level, the mean visual acuity of *rd10* mice (0.31 ± 0.15 and 0.04 ± 0.05 cyc/° at P45 and P164, respectively) was significantly lower than that of WT mice (0.53 ± 0.04 cyc/°) (Figure 3D). Additionally, six *rd10* mice of older ages (three *rd10* mice at P174, one *rd10* mouse at P257, and two *rd10* mice at P259) were tested for visual acuity assessment. Still, no visual acuity was found at any contrast level (data not shown).

### Progressive Decrease in the Ratio of Retinal Ganglion Cells Responding to Light Stimulation in *rd10* Mice

**FIGURE 5:**
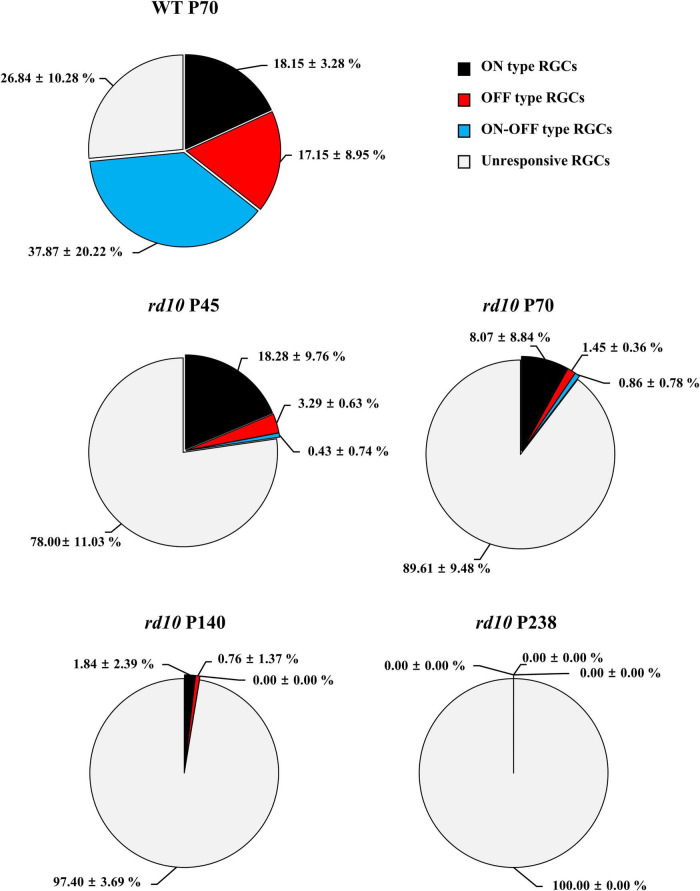
The progressive decrease of retinal ganglion cells (RGCs) that respond to flashed light stimulation in the *rd10* mice. The ON type (black), OFF type (red), ON/OFF type (blue), and unresponsive (light gray) RGCs were counted from isolated retinas. The ON and OFF durations of light stimulation were 4 s, respectively.

Next, we discovered that each RGC subtype divided by light response showed a different disappearance time course according to the postnatal ages. Figure 5 shows the light stimulation-responsive RGCs (LR-RGCs) ratio in WT and *rd10*. We classified LR-RGCs into three groups (ON, OFF, and ON/OFF type) (Masland, 2001; Stasheff, 2008; Stasheff et al., 2011; Baden et al., 2016). Compared with the WT retina, the ratio of LR-RGCs in the P45 *rd10* retina decreased (73% vs. 22%), and with postnatal ages, the ratio of LR-RGCs decreased progressively (22, 10.4, 2.6, and 0%). At P45, the ON type response observed from RD retina was same with WT retina (18.15 ± 3.28% and 18.28 ± 3.28%, p > 0.05), while OFF type (17.15 ± 8.95% and 3.29 ± 0.63%, p < 0.05) and ON/OFF type (37.87 ± 20.22% and 0.43 ± 0.74%, p < 0.05) response significantly decreased. In P238 *rd10* retina, no LR-RGCs were found. The unresponsive RGCs to light stimulus may be due to some RGC subtypes not responding to full-field stimulation. Even in the WT retina, 27% of RGCs belong to this unresponsive group of RGCs.

### Two Response Types of Retinal Ganglion Cells to Electrical Stimulation in *rd10* Mice

**FIGURE 6:**
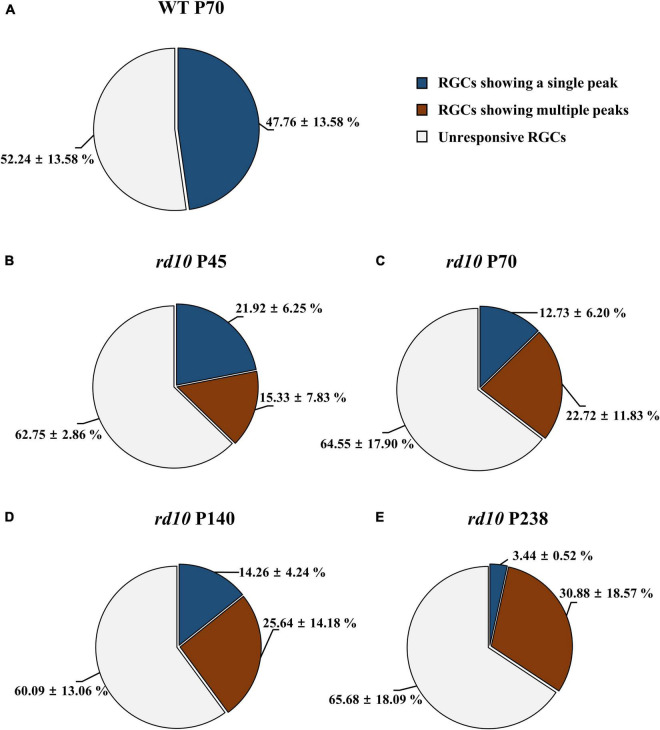
The difference in the ratio of retinal ganglion cells (RGCs) showing multiple peaks among ER-RGCs according to postnatal ages. The ratio of RGCs showing the single peak (blue portion) and multiple peaks (red portions) was calculated in the wild-type (WT) group at P70 **(A)** and *rd10* groups at P45 **(B)**, P70 **(C)**, P140 **(D)**, and P238 **(E)**. The statistics value represents the mean ± standard deviation (SD).

**FIGURE 7:**
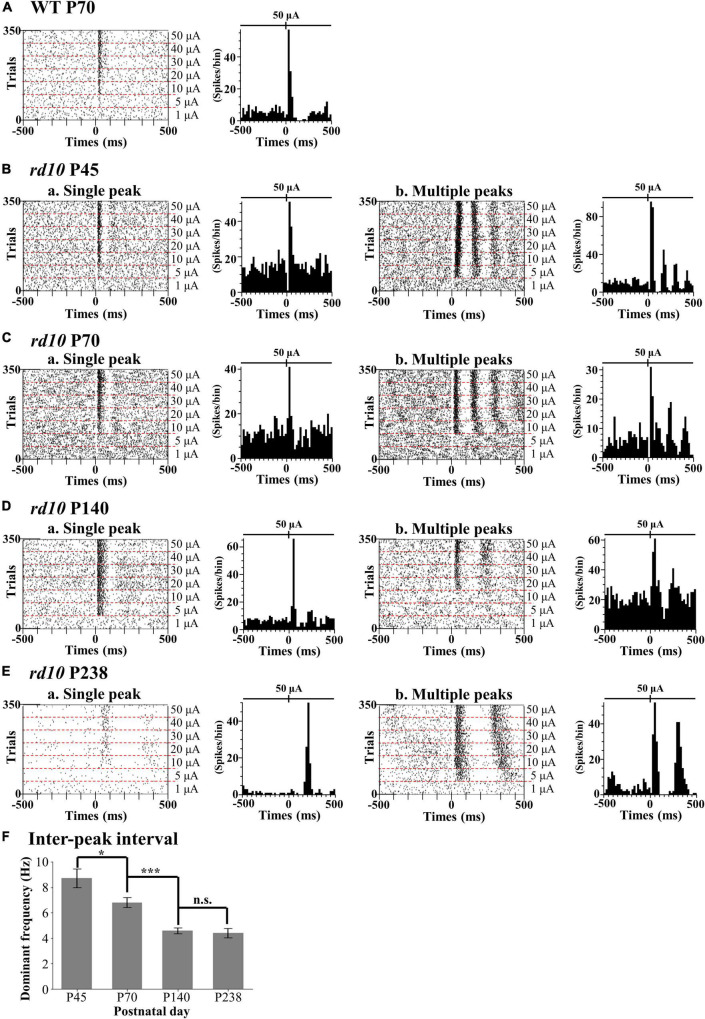
Two different response patterns of the electrical stimulation-responsive retinal ganglion cells (ER-RGCs) according to postnatal ages. Typical response patterns to amplitude-modulated electrical stimulation in the wild-type (WT) group at P70 **(A)** and *rd10* groups at P45 **(B)**, P70 **(C)**, P140 **(D)**, and P238 **(E)** are represented by a raster plot (left panel) and PSTH (right panel). We divided RGCs of *rd10* mice into two groups, single peak (a) and multiple peaks (b) based on the number of peaks in PSTH. The time window of the raster plot and PSTH is set for ±500 ms. The 0 ms represents the time point of stimulus onset. In each raster plot, the stimulation amplitude increases from the bottom to the top row. PSTHs represent electrically evoked RGC response at the strongest pulse amplitude (50 μA). The *y*-axis of PSTH represents the spike number per bin (bin size is 20 ms). **(F)** The inter-peak frequency decreased from P45 to P140 and saturated. The average dominant frequency is shown for each degeneration stage, with error bars representing standard errors. Statistical significance between the average dominant frequencies was calculated for consecutive postnatal days using Student’s *t*-test (***p < 0.001, *p < 0.05, and n.s., *p* > 0.05).

We discovered that the ratio of RGCs showing multiple peaks of spike burst in PSTH increased with postnatal ages in *rd10* mice. We elicited the RGC spikes with electrical stimulation and analyzed the network-mediated RGC response. Like the WT retina, the electrical stimulation-responsive RGCs (ER-RGCs) ratio was observed at ∼40% regardless of the postnatal ages (Figure 6). Figure 7 shows a typical RGC response pattern to amplitude-modulated electrical stimulation. In the WT group, only one response type was observed (Figures 6A, 7A). The electrically evoked spikes formed a single peak within 100 ms from stimulus onset when the pulse amplitude exceeded a threshold level (Figure 7A). However, in the RD group, RGC responses were divided into two types (Figures 6B–E, 7B–E). One response type is a single peak like the WT group; the other response type is multiple peaks (Figures 7B–E).

The inter-peak frequency decreased from P45 to P140 and did not change from P140 to P238 (Figure 7F). At P45, the average dominant frequency was 8.7 Hz (SE = 0.7). At P70, the average dominant frequency was reduced to 6.8 Hz (SE = 0.4). This difference was statistically significant (*p < 0.05 with *t*-test). At P140, the average dominant frequency was further reduced to 4.6 Hz (SE = 0.2). The difference between the average dominant frequencies at P70 and P140 was statistically significant (***p < 0.001 with *t*-test). In contrast, the average dominant frequency at P238 was 4.4 Hz (SE = 0.4). The difference between the average dominant frequencies at P140 and P238 was not statistically significant (n.s., *p* > 0.05).

Multiple peaks occur within 500 ms after the onset of stimulation, but the temporal pattern of occurrence varies according to the postnatal ages. The number of peaks decreased, and the inter-peak intervals increased with older postnatal ages. Moreover, the ratio of RGCs showing multiple peaks among ER-RGCs gradually increased with the postnatal ages (Figures 6B–E). At P45, the number of RGCs showing multiple peaks was 0.70 times smaller than that of RGCs showing a single peak. The number of RGCs showing multiple peaks was 1.78 times, 1.80 times, and 8.98 times larger than that of RGCs showing a single peak, at P70, P140, and, P238, respectively.

**FIGURE 8:**
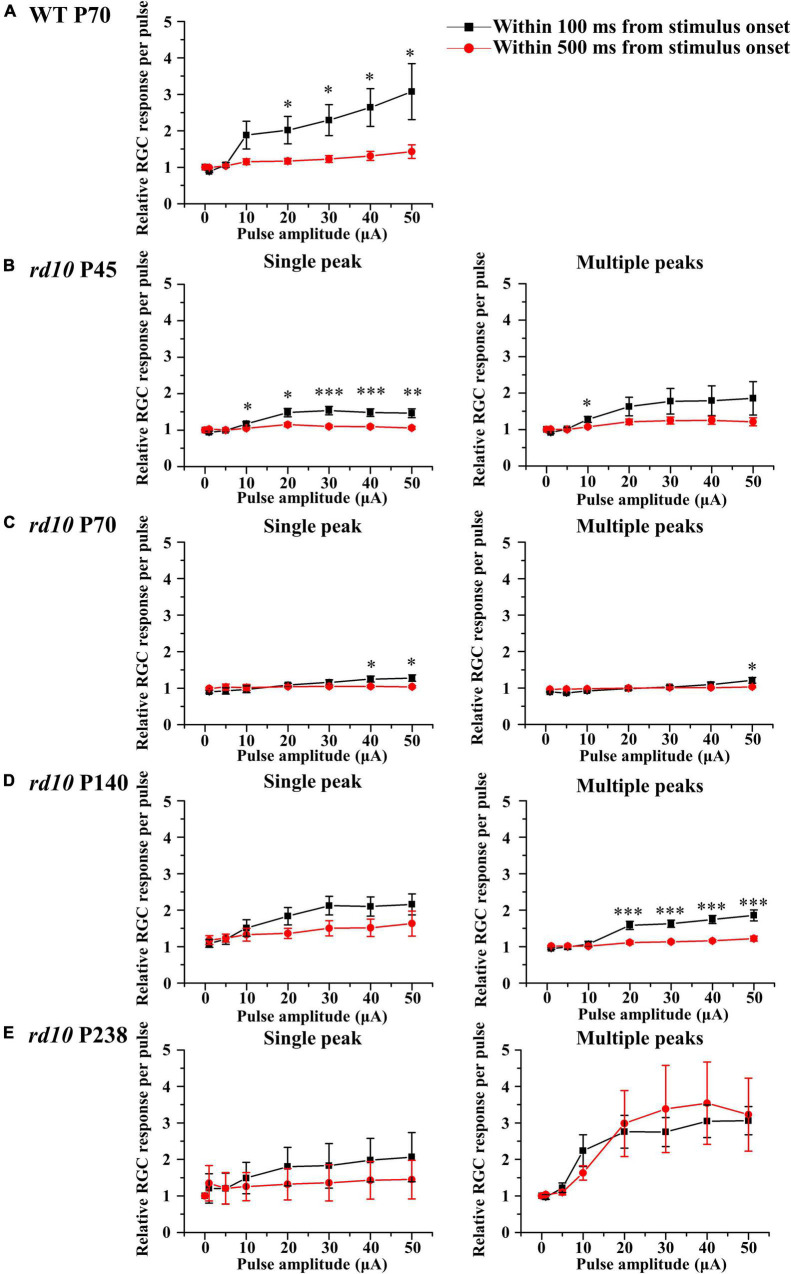
The retinal ganglion cell (RGC) response curves to pulse amplitude modulation for a single peak (left column) or multiple peaks groups (right column). The RGC response curve plotted against pulse amplitude for the WT group at P70 **(A)** and *rd10* groups at P45 **(B)**, P70 **(C)**, P140 **(D)**, and P238 **(E)** groups. Error bars represent the standard error of the mean (SEM). The curves drawn in black include the spikes within 100 ms after stimulus onset, and the curves drawn in red include the spikes within 500 ms after stimulus onset. Asterisks indicate statistical differences between 100 and 500 ms at each pulse amplitude (***p < 0.001, **p < 0.01, and *p < 0.05).

Regardless of multiple peaks, the RGC spikes in *rd10* mice were less elicited by pulse amplitude modulation than in WT mice. Using the average number of spontaneous spikes as a reference, we calculated the relative ratio of the average number of spikes within the 100 or 500 ms from stimulus onset (Figure 8). First, for the group of RGCs showing a single peak, the RGC spikes increased with the increment of pulse amplitude from 10 to 50 μA within 100 ms at P45. However, even if the pulse amplitude increased, the average number of RGC spikes after stimulus onset was similar to that of spontaneous spikes within 500 ms. Second, for the group of RGCs showing multiple peaks, except for the *rd10* P238 group, the response curves within 100 and 500 ms were similar to those of RGCs showing a single peak. However, in the *rd10* P238 group, the response curves within 100 ms as well as within 500 ms showed a linear relationship according to pulse amplitude increase.

## Discussion

### Validation of Newly Established *rd10* Mice (C57BL/6-*Pde6b^em1(R560C)Dkl^*/Korl)

The occurrence rate of RP is lower than that of other retinopathies (Cheung et al., 2010; Wong et al., 2014; Rim et al., 2017). In people aged 20–74 years, diabetic retinopathy (DR) prevalence is 8% in the United States and 10% in Southeast Asia (Cheung et al., 2010). The occurrence rate of RP is six cases/100,000 persons⋅year in the United States and 1.64 cases/100,000 persons⋅year in South Korea, regardless of age (Rim et al., 2017). However, RP is a monogenic disease, while DR and age-related macular degeneration (AMD) are chronic and polygenic diseases (Bowne et al., 2011; Ratnapriya and Chew, 2013; Cho and Sobrin, 2014). Therefore, in establishing an animal model, RP has advantages over DR or AMD since it requires an introduction of a single mutation into a single gene and the phenotype appears quickly at a younger age than DR or AMD.

The *rd1* and *rd10* mice are RP models with the pde6b gene mutations. The *rd1* mouse has a nonsense mutation, while the *rd10* mouse has a missense mutation (Chang et al., 2002, 2007). This difference causes delayed onset and slower progression of RD in the *rd10* mouse compared with the *rd1* mouse. The progressive death of rod photoreceptors appears from P16 to P60 in the *rd10* mouse, while it appears from P8 to P20 in the *rd1* mice (Pennesi et al., 2012). Thus, the *rd10* mouse can provide a wider therapeutic window than the *rd1* mouse.

In this study, we used the *rd10* mouse strain (C57BL/6-*Pde6b^em1(R560C)Dkl^*/Korl) newly established by the National Institute of Food and Drug Safety Evaluation (NIFDS) of South Korea instead of the conventional *rd10* mouse strain (B6.CXB1-*Pde6b^rd10^*/J) from Jackson Lab (Bar Harbor, ME, United States). We validated the histological and behavioral phenotype of the newly established *rd10* strain.

The histological findings in our study showed the progressive disappearance of the fluorescence signals of rhodopsin in rods and opsin in cones until P45 and P70, respectively. We also showed that a single row of nuclei was maintained in the ONL from P45 to P238 (Figure 4). In *rd10* mice, RD begins at P18–P20, and rod photoreceptor loss lasts until P45 (Chang et al., 2007; Gargini et al., 2007; Barhoum et al., 2008; Stasheff et al., 2011; Pennesi et al., 2012; Li et al., 2019). The cone photoreceptors disappear by 2 months after birth (∼P60), but the single nuclear layer of the ONL persists until 9 months after birth (∼P270) (Gargini et al., 2007). Our histological results are consistent with the literature.

Through behavioral experiments, we also show that the visual acuity of the newly established *rd10* strain is lower than that of WT mice. The measured visual acuity of WT mice at P70–P71 and *rd10* mice at P45–P47 and P164–P165 was 0.53 ± 0.04 cyc/°, 0.31 ± 0.15 cyc/°, and 0.04 ± 0.05 cyc/°, respectively (Figure 3). These behavioral phenotypes of our study and previous studies are compatible (Benkner et al., 2013; Vagni et al., 2019). In an earlier study using the Optodrum, for WT mice on P59–P63 and conventional *rd10* mice on P24–P32 and P86–91, the visual acuity of WT and *rd10* mice was 0.38 ± 0.005 cyc/°, 0.35 ± 0.009 cyc/°, and 0.2 cyc/°, respectively (Benkner et al., 2013). In a previous study using a different system for detecting an optomotor response from us, the visual acuity of WT and conventional *rd10* mice was 0.40 ± 0.01 cyc/° and 0.12 ± 0.01 cyc/°, respectively, at P30 (Vagni et al., 2019). In addition, they also showed that the visual acuity of conventional *rd10* mice strain declines with postnatal ages from P30 to P60 while WT mice maintain visual acuity until P60. Our results of the behavioral study are consistent with the literature.

In summary, the newly established *rd10* mice strain (C57BL/6-*Pde6b^em1(R560C)Dkl^*/Korl) shows a histological and behavioral phenotype similar to that of the conventional *rd10* mice strain (B6.CXB1-*Pde6b^rd10^*/J). Therefore, we believe that newly established *rd10* mice are as reliable as conventional *rd10* mice for RD animal models.

### The ON Response Preserves Longer Than the OFF Response in *rd10* Mice With RD

We found the RGC type-dependent degeneration in *rd10* mice. The ON response sustained longer than the OFF response with RD. The OFF response quickly disappeared, while the ON response was preserved at P45. The ON response slowly disappeared until P140 (Figure 5). This study detected an average of 72% of LR-RGCs per patch from WT retinas with simple full-field illumination. Our experimental condition of light stimulation (OFF luminance ≈ 5.63 cd/m^2^ and ON luminance ≈ 482.18 cd/m^2^) is mesopic, where both rod and cone photoreceptors respond to light stimulation (Tikidji-Hamburyan et al., 2017; Uchida et al., 2018).

We classified the LR-RGCs into three subtypes: ON, OFF, and ON/OFF (18, 17, and 38%, respectively). Even in the WT retina, 27% of RGCs belong to an unresponsive group of RGCs. The unresponsive RGCs to light stimulus may be due to some RGC subtypes not responding to full-field stimulation. As is well known, an RGC has a center-surround receptive field (Turner et al., 2018). If the center and surround of the receptive field are simultaneously stimulated, the RGC may not fire spikes due to center-surround antagonism. It was reported that full-field stimulation significantly reduced both the peak and the mean firing rates by 60% compared to an optimal spot stimulus (Sagdullaev and McCall, 2005). However, in our experimental setup with MEA, full-field stimulation still induced the light response in a substantial amount of RGCs (∼73%) (Figure 5). In addition, there is a possibility that some unresponsive cells are displaced spiking-amacrine cells (ACs). In the mouse retina, around 37–59% of the displaced ACs exist in the GCL (Muller et al., 2010; Baden et al., 2016). The spiking ACs in the GCL are reported to respond to light stimulation because they are coupled with ipRGCs *via* gap junction (Reifler et al., 2015). However, to the authors’ best knowledge, there is no report showing how many percentages of displaced ACs comprise the spiking ACs.

As the retina degenerates, the ratio of LR-RGCs progressively decreases until P140 and LR-RGCs completely disappear at P238 (Figure 5). One possible explanation for this reduced ratio of LR-RGCs between P70 and P140 is the second-stage degeneration of cone-cell death. The cone-cell death has been explained mainly through necroptosis and partly by receptor-interacting protein (RIP) kinase. The necroptosis occurs due to neurovascular remodeling. The neurovascular remodeling involves the blood–retina barrier (BRB) leak, the glial sealing in the choroid, and the displacement of the retinal pigment epithelium (RPE) cells (Murakami et al., 2012; Ivanova et al., 2019).

**FIGURE 9:**
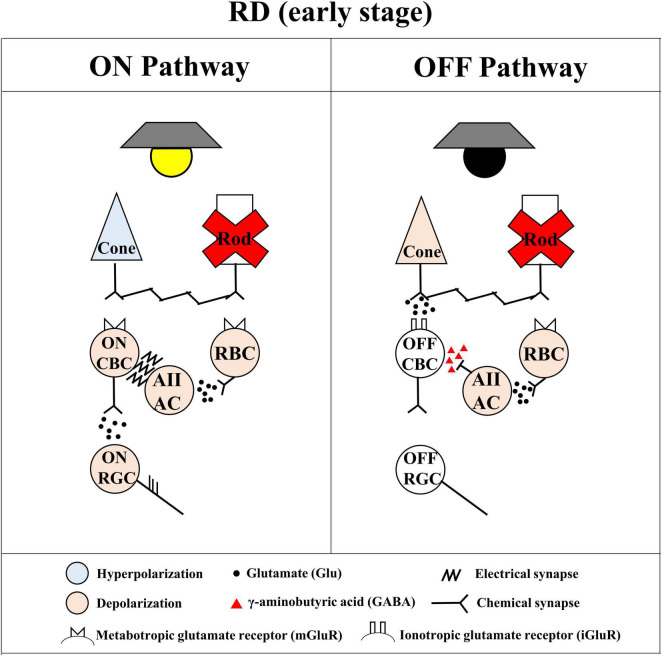
Simple diagram of spiking pathway of ON and OFF retinal ganglion cells (RGCs) in the retinal degeneration (RD) to the mesopic light stimulation. The simple diagram of ON and OFF pathways in the degenerated retina. The stimulated cone photoreceptors affect both ON cone bipolar cells (ON CBCs) and OFF cone bipolar cells (OFF CBCs) *via* the metabotropic glutamate receptor (mGluR) and the ionotropic glutamate receptor (iGluR)-mediated chemical synapse, respectively. The ON and OFF CBCs directly connect with ON and OFF RGCs, respectively, generating their spikes with glutamatergic input. On the other hand, the stimulated rod photoreceptors affect only rod bipolar cells (RBCs) *via* the mGluR. The RBCs indirectly connect with ON and OFF RGCs through AII ACs. The AII ACs synapse with ON and OFF RGCs via the gap junction channel-mediated electrical synapse and GABAergic synapse, respectively. In the case of RD, the rod pathway cannot show electro-pharmacological alteration even if the OFF light stimulation activates the OFF pathway.

The cause of the distinct difference between ON and OFF RGC responses could be explained by the different electro-pharmacological properties between ON and OFF pathways. The preservation of the GCL even after RD (Figure 4B) implies that the disappearance of OFF RGC response might be due to the reduced firing of OFF RGC, not due to the death of OFF RGCs. Therefore, we postulated the following hypothesis (Figure 9). The ON and OFF pathways in the normal retina consist of cone and rod pathways (Davanger et al., 1991; Wettschureck and Offermanns, 2005; Volgyi et al., 2013; Fain and Sampath, 2018). In the cone pathway, cone photoreceptors synapse with ON cone bipolar cells (ON CBCs) and OFF cone bipolar cells (OFF CBCs). ON CBCs and OFF CBCs directly synapse ON RGCs and OFF RGCs, respectively.

On the other hand, in the rod pathway, rod photoreceptors only synapse with RBCs. The RBCs indirectly connect with ON and OFF RGCs through AII ACs. In the *rd10* mice retina, the loss of rod photoreceptors (Figure 4) causes the subsequent decrease of glutamate concentration to RBCs. As a result, the membrane potential of RBCs and AII ACs will be depolarized and maintained regardless of ON and OFF stimulations. Consequently, the γ-aminobutyric acid (GABA) released from AII ACs to OFF CBC will inhibit the OFF response. On the contrary, the electrical synapse between AII AC and ON CBC might be enhanced, then ON RGCs are likely to fire spikes.

### The Electrical Stimulation Hardly Elicits Retinal Ganglion Cells Spikes in Retinal Degeneration Compared With Wild-Type

Our results showed that RGCs in the RD retina produced fewer spikes than in the WT retina (Figure 8). *In vitro* studies using the mouse, rat, and pig models reported that fewer RGC spikes are evoked in the RD retina than in the WT retina with electrical stimulation (Goo et al., 2011b; Jensen, 2012; Cha et al., 2021). *In vivo* studies in human subjects also showed that higher stimulus intensity was required to induce phosphenes in patients with RP than in people with normal vision (Delbeke et al., 2001; Gekeler et al., 2006).

The difficulty in eliciting RGC spikes in the degenerate retina may be explained by spontaneous hyperactivity due to oscillations and multiple peaks of spike bursts of RGCs. As RD progresses, loss of photoreceptors leads to retinal remodeling in the INL (Jones et al., 2003; Marc et al., 2003; Jones et al., 2012), resulting in aberrant oscillation (frequency 5∼10 Hz) through gap junction coupling between AII AC and ON CBC (Yee et al., 2012; Trenholm and Awatramani, 2015). Oscillations induce spontaneous hyperactivity with multiple bursts of RGCs, requiring a higher stimulation threshold for RGC activation (Margolis et al., 2008; Stasheff, 2008; Yee et al., 2012; Goo et al., 2016). Interestingly, the multiple bursts observed during electrical stimulation are also found in optogenetic stimulation (Reh et al., 2022). When Channelrhodopsin-2 expressed in RBCs of *rd10* mice (P182-215) was stimulated optogenetically, RGCs showed multiple bursts. No multiple bursts were found when local stimulation was applied to the retina, whereas multiple bursts were observed when larger retinal areas were stimulated. This finding suggests the possibility of large-scale network activation by gap junction coupling between AII AC and ON CBC. Recent studies support this hypothesis by showing that the stimulation efficiency is enhanced when oscillation and multiple bursts are abolished by gap junction blockers such as meclofenamic acid (Eleftheriou et al., 2017; Ahn et al., 2022). This study and our previous report (Goo et al., 2016) showed that the number of *rd10* RGCs showing multiple bursts increased by the postnatal day (P45–238) (Figure 6). Therefore, multiple bursts might be regarded as a physiological indicator in RD. In the future, instead of a conventional hardware-based approach such as increasing the number of electrodes in the prosthesis, the suppression of oscillation and multiple bursts could be an excellent strategy to improve the visual performance of retinal prosthesis.

Another possibility that RGC spikes are hardly elicited by electrical stimulation in the degenerated retina is shielding the electrical pulse to RGC by the Müller cell. As we showed in Figure 4A, as direct evidence of glia sealing in the degenerated retina after P45, Müller cell hypertrophy is detected through the glutamine synthetase (GS) signal. However, to show Müller cell hypertrophy, GFAP staining for Müller cell endfeet would be a better option than GS staining for Müller cell soma we used (Gargini et al., 2007). In future work, GFAP staining for Müller cell endfeet is planned. Several studies using various RD animals such as the mouse, rat, rabbit, pig, and human also reported the enclosure of RGCs by the Müller cell (Jones et al., 2003; Bringmann et al., 2006; Gargini et al., 2007; Wan et al., 2008; Ahn et al., 2019; Choi et al., 2021). The shielding by the Müller cell prevents BCs or RGCs from being stimulated by a 2D electrode (Aplin et al., 2016). By using 3D electrodes to get closer to BC or RGC (Airaghi Leccardi et al., 2019; Davidsen et al., 2019; Ho et al., 2019; Seo et al., 2019; Huang et al., 2021), we would expect to elicit more spikes under the same stimulus conditions with this study.

### Progression of the Retinal Degeneration Causes the Increase in the Inter-peak Interval of Retinal Ganglion Cells to Electrical Stimulation

Our results showed that the inter-peak (inter-burst) interval of *rd10* RGCs increased with the postnatal ages (Figure 7F). The increase in the inter-peak interval with the progression of RD could be explained by a decrease in gap junction coupling between ACs and ON cone BCs. Gap junctions are essential for generating abnormal rhythmic bursts and oscillations in *rd10* RGCs (Toychiev et al., 2013; Menzler et al., 2014; Ivanova et al., 2016). In particular, it is well known that the gap junction coupling between AII ACs and ON-cone BCs is deeply involved in oscillation generation (Trenholm et al., 2012; Yee et al., 2012; Choi et al., 2014). Gap junction blocking with meclofenamic acid (MFA) induces a lower oscillation frequency (Choi et al., 2014). Therefore, the increase in the inter-peak interval (lower frequency of oscillation) of RGCs at P238 shown in Figure 7F may be associated with a decrease in gap junction coupling as RD progresses.

The other possibility is that the decrease in inhibitory input results in an increased inter-peak interval of RGCs observed in this study (Figure 7). Studies show that inhibitory input modulates the oscillation frequency (Ye and Goo, 2007; Biswas et al., 2014). The oscillations did not disappear when *rd10* retinas were treated with GABA and glycine receptor inhibitors (bicuculine and strychnine, respectively), but the oscillation frequency decreased from ∼6 to ∼1 Hz. Indeed, previous histological findings seem to support this hypothesis. In *rd10* mice, the number of horizontal cells, one of the inhibitory retinal neurons, decreased significantly over time, approximately 19% between P42 and P98 and 29% at P252 (Gargini et al., 2007). Therefore, the gradual increase in the inter-peak interval of RGCs (lower frequency of oscillation) from P45 to P238 observed in this study may be closely related to the decrease in inhibitory input due to the reduction in the horizontal cell number during RD.

### Efficiency of Electrical Stimulation Regarding the Presence of Multiple Peaks in Retinal Degeneration Retina

Unlike other age groups of *rd10* mice, RGCs showing multiple peaks of spike burst in the late stage of RD at P238 responded well to the electrical stimulation (Figure 8E). To quantify the electrically evoked RGC response, we calculated the mean firing rate within 100 or 500 ms from stimulus onset. The response profile of RGCs shows that a single peak appears within 100 ms from stimulus onset for both WT and RD. The first peak of multiple peaks also appears within 100 ms from stimulus onset in the RD. The other peaks of multiple peaks appear within 500 ms from stimulus onset. In RGCs showing a single peak from the WT and RD, due to spontaneously generated spikes within 100 to 500 ms, the mean firing rate within 500 ms is similar to the spontaneous firing rate (Figures 8A–E, left). The relative ratio of RGC response sticks to the value of 1.0 in the RD except for P238; the mean firing rate within 500 ms is similar to the spontaneous firing rate (Figures 8B–D, right). Our previous study reported that pulse amplitudes significantly modulate only the first and second peaks despite three and more multiple peaks appearing (Ryu et al., 2010). The group of RGCs in P238 has only two peaks within 500 ms from stimulus onset, while RGCs in the other age groups have third and more peaks within 500 ms from stimulus onset. The difference in time window showing first and second peaks at different degeneration stages could explain the flat RGC response curve with pulse amplitude modulation in the earlier stage of RD at P45 to P140 than P238. However, there are limited reports regarding the relationship between multiple peaks’ presence and electrical stimulation’s efficiency. Therefore, in the future, we are planning to explore this issue.

### Clinical Implication and Future Work

To develop reliable retinal prostheses, we should find ways to effectively elicit RGC spikes with electrical stimulation (Stingl et al., 2017; Ayton et al., 2020; Delyfer et al., 2021; Yoon et al., 2021). The electrical stimulation elicits two different RGC spike responses according to the prosthesis location in the retina (Margalit et al., 2002; Zrenner, 2002; Ayton et al., 2020; Im and Kim, 2020). One is the direct response originating in RGCs by epi-retinal stimulation configuration. The other is the network-mediated response originating in BCs or photoreceptors by sub-retinal stimulation configuration (Boinagrov et al., 2014). While the direct response has the advantage of response modulation by direct matching an RGC spike and a stimulus pulse, the network-mediated response has the advantage of mimicking the natural RGC response *via* the retinal network (Zrenner, 2002; Fried et al., 2006; Im and Fried, 2015; Im and Kim, 2020). Despite many efforts with retinal prostheses, regardless of their stimulus configuration, the artificial vision showed low spatial resolution performance below the 20/420 of visual acuity (Ayton et al., 2020; Im and Kim, 2020).

In the artificial vision research community, the main interests are how ON RGC and OFF RGC react to electrical stimulation and how efficiently and selectively ON or OFF RGC responses are activated. In literature, many efforts have been undertaken for this. As one of the stimulation strategies for improving the spatial resolution of retinal prostheses, selective activation of individual RGCs has been attempted (Tong et al., 2020). In direct response, selective activation of ON or OFF RGCs *via* pulse amplitude modulation and frequency modulation is possible in the WT retina (Twyford et al., 2014; Guo et al., 2018; Kotsakidis et al., 2018). While OFF RGCs preferentially respond to the lower pulse amplitude (<90 μA) regardless of stimulus frequency, ON RGCs preferentially respond to the higher pulse amplitude (>150 μA) and stimulus frequency (>2 kHz) (Tong et al., 2020). On the other hand, the electrically evoked network-mediated spikes are differently induced according to the RGC subtype *via* the pulse duration modulation in the WT retina (Im and Fried, 2015, 2016; Im et al., 2018). The ON RGC showed strong correlations between light-evoked spikes and electrically evoked network-mediated spikes but not the OFF RGC (Im and Fried, 2015). The repetitive stimulation leads to a reset of the ON RGC, but it leads to desensitization of the OFF RGC (Im and Fried, 2016). The electrically evoked spikes of ON RGC were well-modulated to stimulus duration than the OFF RGC (Im et al., 2018). The ON RGC shows a gradual decline in peak firing rate and inter-trial correlation until P60 in *rd10* mice, but not the OFF RGC (Yoon et al., 2020). In addition, regarding the reliability of electrically evoked RGC response, ON RGC shows a gradual increment of fano factor until P60, but not the OFF RGC (Yoon et al., 2020). Our result with light stimulation shows that the ON response preserves longer than the OFF response in the early stage of RD (Figure 5). In our result with electrical stimulation, the network-mediated responses of OFF RGCs are likely to be elicited less than ON RGCs by the aberrant rod pathway in the RD (Figure 9). Like the previous reports, our result supports the electrically evoked network-mediated RGC responses favor ON RGCs. All these studies have been performed in explanted *in vitro* retinas. ON and OFF RGCs respond to opposite brightness polarities with natural light stimulation, but ON and OFF RGCs are stimulated simultaneously with electrical stimulation. To the best knowledge of the authors, there is no report for the selective activation of ON or OFF RGCs in retinal prosthesis implanted patients.

Electrical stimulation induces simultaneous excitation of ON and OFF RGC, which leads to the cancelation of signals in higher visual centers of the cortex (Kotsakidis et al., 2018). The optogenetic approach, a different approach for vision restoration, has been proposed to overcome this limitation of electrical stimulation. The optogenetic approach using channel rhodopsin has been proposed for the selective activation of ON or OFF RGCs (Provansal et al., 2022). Optogenetic therapy using ChrimsonR in the living primate (*Macaca fascicularis*) is reported to restore characteristic RGC responses to patterned stimuli (McGregor et al., 2020, 2022). In a clinical trial, an optogenetic approach using AAV2.7m8-CAG-ChrimsonR-tdTomato for one patient with RP showed an excellent success rate increment with objective visual detection task (before and after optogenetic approach: 5.8% vs. 41%, respectively) (Sahel et al., 2021). Even if this clinical success is from only one patient, this is a very promising result. The abovementioned ChrimsonR-mediated restoration is mediated by ON RGC response. However, in physiology, ON or OFF characteristics starts from ON BC or OFF BC not from ON RGC or OFF RGC. Therefore, selective activation of ON BC or OFF BC could be a good candidate for an optogenetic approach in the future.

In terms of using a natural visual pathway, the gene therapy that prevents the progression of RD or restores degenerated photoreceptors may also be an alternative to electrical stimulation. The gene therapy approach has been proposed to deliver WT copies of the defective gene into the photoreceptor of the RD. The CRISPR-Cas9 with adeno-associated viral (AAV) vector offers a viable strategy for restoring visual function in the RD mouse model (Pang et al., 2008; Moreno et al., 2018; Vagni et al., 2019; Nishiguchi et al., 2020). Supplementation of *Pde6b* (Pang et al., 2008; Vagni et al., 2019) or repression of *Nrl* (Moreno et al., 2018) in P7-14 *rd10* mice lead to delayed degeneration in comparison with untreated age-matched *rd10* mice. Supplementation of *Gnat1* also leads to delayed degeneration in *Pde6c*^cpfl1/cpfl1^*Gnat1*^IRD2/IRD2^** mice (Nishiguchi et al., 2020).

## Conclusion

This study observed the histological, behavioral, and electrophysiological phenotypes with degeneration stages in *rd10* mice. First, we validated age-dependent histological changes in the retina and behavioral changes in the optomotor response in the newly established *rd10* mice (C57BL/6-*Pde6b^em1(R560C)Dkl^*/Korl). Therefore, the newly established *rd10* mice strain could be as reliably used as the conventional *rd10* mice strain (B6.CXB1-*Pde6b^rd10^*/J) for the animal model for RP. Second, we investigated RGC response to light or electrical stimulations according to degeneration stages. By the light stimulus, the ON response sustains longer than the OFF response in the early stage of RD (P45). The *rd10* RGC generates fewer spikes than WT throughout the degeneration stages despite the same electrical stimulation parameters. With the progress of the degeneration stage, the number of RGCs which display PSTHs with multiple peaks increases. In addition, the electrically evoked RGC spikes by the pulse amplitude modulation differ across postnatal ages. Therefore, we suggest optimizing the stimulation protocol, such as nullifying multiple peaks, could benefit better clinical results.

## Data Availability Statement

The raw data supporting the conclusions of this article will be made available by the authors, without undue reservation.

## Ethics Statement

The animal study was reviewed and approved by the Institutional Animal Care and Use Committee of the Chungbuk National University (approval number: CBNUA-1520-21-01).

## Author Contributions

SC, YHL, HKK, and YSG conceived the study. DL generated the newly established rd10 mouse (C57BL/6-*Pde6b^em1(R560C)Dkl^*/Korl) and interpreted data for the work. SC and YJ performed the experiments. SC, YJ, and JA analyzed the data. SC, JA, YY, and YSG wrote the manuscript. All authors contributed to the article and approved the submitted version.

## Conflict of Interest

The authors declare that the research was conducted in the absence of any commercial or financial relationships that could be construed as a potential conflict of interest.

## Publisher’s Note

All claims expressed in this article are solely those of the authors and do not necessarily represent those of their affiliated organizations, or those of the publisher, the editors and the reviewers. Any product that may be evaluated in this article, or claim that may be made by its manufacturer, is not guaranteed or endorsed by the publisher.

## Supplementary Material

Supplementary Figure 1 Generation of *Pde6b* knockin mouse using CRISPR-Cas9. **(A)** A schematic illustration showing the location of the *Pde6b* target gRNA, HhaI restriction sites, and primer binding sites along with C to T mutation point. **(B)** Initial screening of F0 *Pde6b* knockin mutants with PCR genotyping. Digestion of PCR products with HhaI yielded 3, 4, and 2 DNA fragments in wild type (+/+), heterozygote (*+/*M), and homozygote mutant (M/M) samples, respectively. **(C)** The exact sequence of knockin mutation was verified by sequencing after TA cloning of PCR product from F1 heterozygotes mice. The desired knockin of mutation R560C (red arrow) with extra 4 silence mutations (black arrows) are shown.

Supplementary Figure 2 Example of the raster plot and the PSTH of light stimulus-responsive RGCs. The raster plot shows the time stamp of RGC spikes for each trial. The PSTH shows cumulated spike counts per bin for 50 trials of light stimulation (bin size is 20 ms). The bars above the raster plot represent the 4 s light onset and light offset. The ON and OFF RGCs shown include both the brisk transient (BT) and brisk sustained (BS) RGCs.
