# Supplementary material for: Stage-Dependent Changes of Visual Function and Electrical Response of the Retina in the rd10 Mouse Model
Source: Front Cell Neurosci. 2022 Jul 19;16:926096. doi: 10.3389/fncel.2022.926096 (PMC9345760; doi:10.3389/fncel.2022.926096)
Supplement: Supplementary Figure 1 — Generation of Pde6b knockin mouse using CRISPR-Cas9. (A) A schematic illustration showing the location of the Pde6b target gRNA, HhaI restriction sites, and primer binding sites along with C to T mutation point. (B) Initial screening of F0 Pde6b knockin mutants with PCR genotyping. Digestion of PCR products with HhaI yielded 3, 4, and 2 DNA fragments in wild type (+/+), heterozygote (+/M), and homozygote mutant (M/M) samples, respectively. (C) The exact sequence of knockin mutation was verified by sequencing after TA cloning of PCR product from F1 heterozygotes mice. The desired knockin of mutation R560C (red arrow) with extra 4 silence mutations (black arrows) are shown. [file Data_Sheet_1.docx]

**
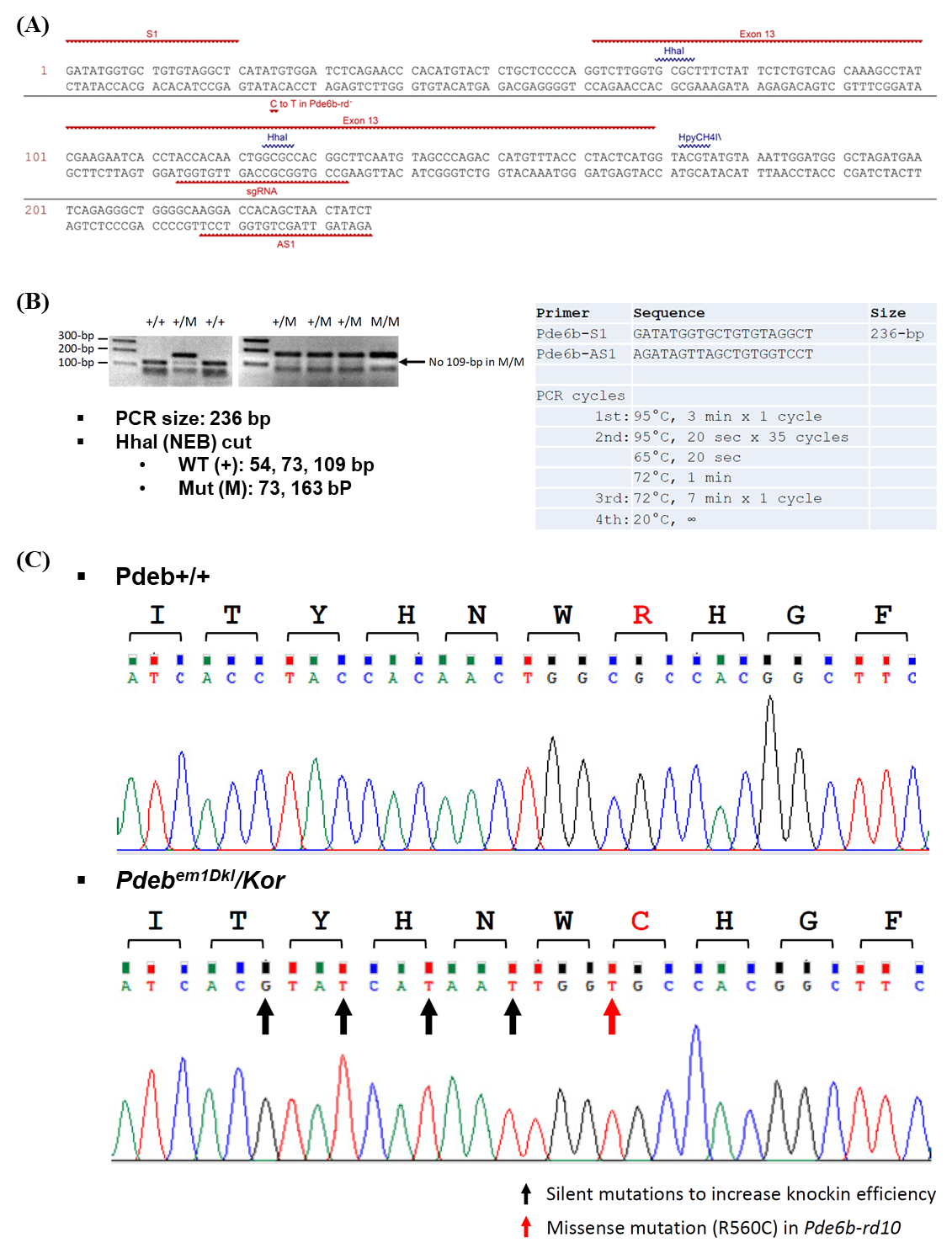
**

**Supplementary Figure 1. Generation of *Pde6b* knockin mouse using CRISPR-Cas9. (A)** A schematic illustration showing the location of the *Pde6b* target gRNA, HhaI restriction sites, and primer binding sites along with C to T mutation point. **(B)** Initial screening of F0 *Pde6b* knockin mutants with PCR genotyping. Digestion of PCR products with HhaI yielded 3, 4 and 2 DNA fragments in wild type (+/+), heterozygote (*+/*M), and homozygote mutant (M/M) samples, respectively. **(C)** The exact sequence of knockin mutation was verified by sequencing after TA cloning of PCR product from F1 heterozygotes mice. The desired knockin of mutation R560C (red arrow) with extra 4 silence mutations (black arrows) are shown.

**
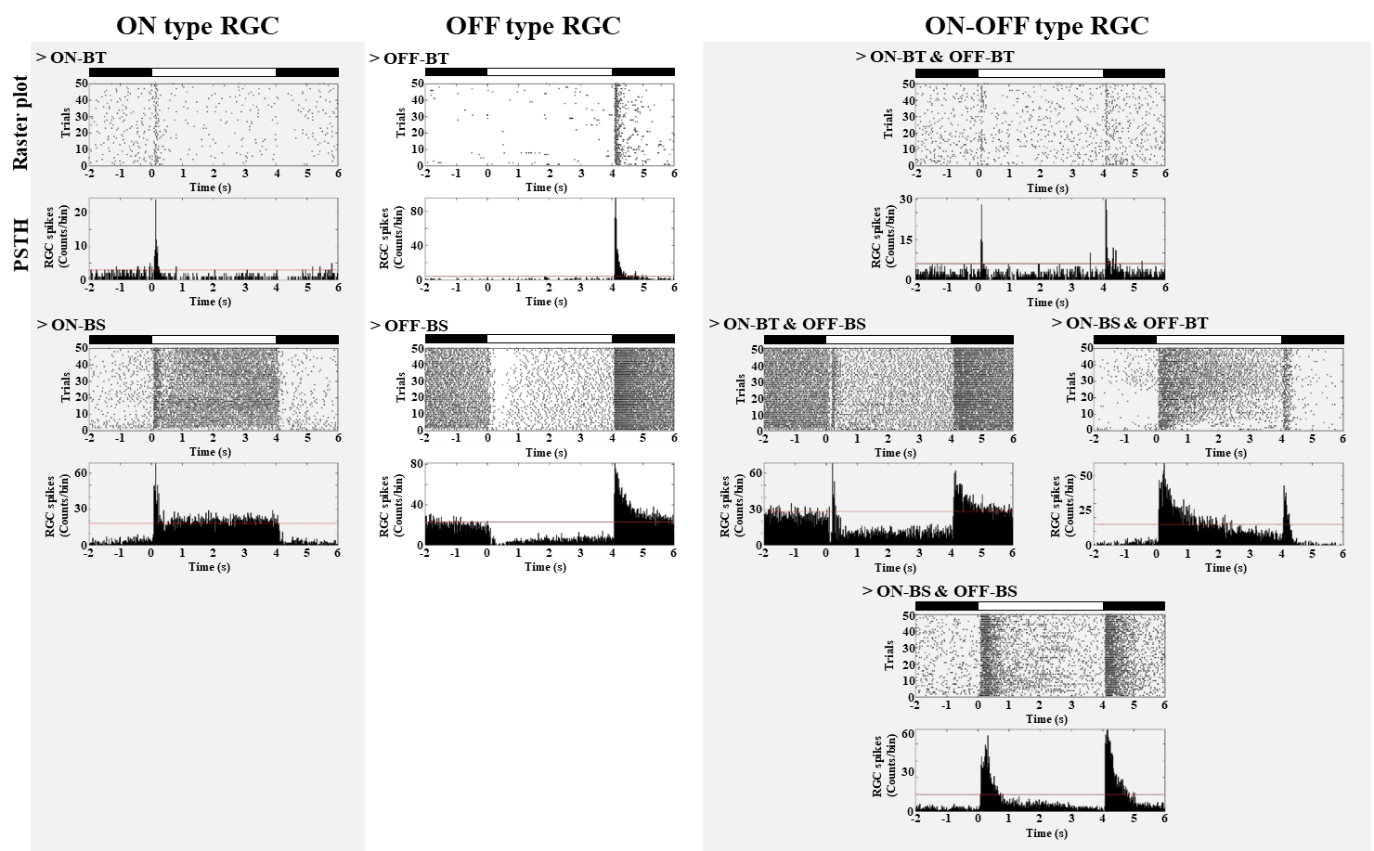
**

**Supplementary Figure 2. Example of the raster plot and the PSTH of light stimulus-responsive RGCs.** The raster plot shows the time stamp of RGC spikes for each trial. The PSTH shows cumulated spike counts per bin for 50 trials of light stimulation (bin size is 20 ms). The bars above the raster plot represent the 4 sec light onset and light offset. The ON and OFF RGCs shown include both the brisk transient (BT) and brisk sustained (BS) RGCs.
